# Towards a mechanistic understanding of lipodystrophy and seipin functions

**DOI:** 10.1042/BSR20140114

**Published:** 2014-10-02

**Authors:** Kenneth Wee, Wulin Yang, Shigeki Sugii, Weiping Han

**Affiliations:** *Singapore Bioimaging Consortium, Agency for Science, Technology and Research (A*STAR), Singapore; †Department of Biochemistry, Yong Loo Lin School of Medicine, National University of Singapore, Singapore; ‡Cardiovascular and Metabolic Disorders Program, Duke-NUS Graduate Medical School, Singapore

**Keywords:** adipocyte, lipid droplet, lipin, lipolysis, metabolism, obesity, AGPAT, 1-acylglycerol-3-phosphate-O-acyl-transferase, BSCL, Berardinelli–Seip congenital lipodystrophy, C/EBP, CCAAT/enhancer binding protein, CANDLE, chronic atypical neutrophilic dermatosis with lipodystrophy and elevated temperature, Cav1, Caveolin-1, CGL, congenital generalized lipodystrophy, ER, endoplasmic reticulum, HCV, hepatitis C virus, IL-6, interleukin-6, LD, lipid droplet, LPA, lysophosphatidic acid, MEF, mouse embryonic fibroblasts, NFAT, nuclear factor of activated T cells, nSREBP1c, nuclear SREBP1c, PA, phosphatidic acid, Pio, Pioglitazone, PKA, protein kinase A, PPARγ, peroxisome proliferator-activated receptor gamma, PTRF, polymerase I and transcript release factor, TAG, triacylglycerol, TMDs, transmembrane domains, TNFα, tumor necrosis factor alpha, WAT, white adipose tissue

## Abstract

CGL (Congenital generalized lipodystrophy) is a genetic disorder characterized by near complete loss of adipose tissue along with increased ectopic fat storage in other organs including liver and muscle. Of the four CGL types, BSCL2 (Berardinelli–Seip Congenital lipodystrophy type 2), resulting from mutations in the *BSCL2/seipin* gene, exhibits the most severe lipodystrophic phenotype with loss of both metabolic and mechanical adipose depots. The majority of *Seipin* mutations cause C-terminal truncations, along with a handful of point mutations. Seipin localizes to the ER and is composed of a conserved region including a luminal loop and two transmembrane domains, plus cytosolic N- and C-termini. Animal models deficient in seipin recapitulate the human lipodystrophic phenotype. Cells isolated from seipin knockout mouse models also exhibit impaired adipogenesis. Mechanistically, seipin appears to function as a scaffolding protein to bring together interacting partners essential for lipid metabolism and LD (lipid droplet) formation during adipocyte development. Moreover, cell line and genetic studies indicate that seipin functions in a cell-autonomous manner. Here we will provide a brief overview of the genetic association of the CGLs, and focus on the current understanding of differential contributions of distinct seipin domains to lipid storage and adipogenesis. We will also discuss the roles of seipin-interacting partners, including lipin 1 and 14-3-3β, in mediating seipin-dependent regulation of cellular pathways such as actin cytoskeletal remodelling.

## INTRODUCTION

Lipodystrophy is characterized by the selective loss of adipose tissue, with manifestation of clinically heterogeneous phenotypes. Since a key function of adipose tissue is the storage of excess energy in the form of lipids, extreme loss of fat in lipodystrophy results in hypertriglyceridemia and ectopic accumulation of TAG (triacylglycerol) in other organs, particularly liver, leading to a severe metabolic condition. Not surprisingly, lipodystrophy is often accompanied by insulin resistance and its complications of diabetes mellitus, hepatic steatosis, acanthosis nigricans and dyslipidaemia [1]. Other major manifestations include muscular hypertrophy and alterations of the reproductive system (increase in external genitalia size) and renal disorder [2]. There are two types of lipodystrophy: acquired and inherited. Acquired lipodystrophy, more common than inherited lipodystrophy, is not genetically predisposed, and can be categorized as HIV-infected lipodystrophy, acquired partial lipodystrophy, acquired generalized lipodystrophy and localized lipodystrophy. Clinical features include the aforementioned as well as low levels of leptin and adiponectin in serum [1,3]. Inherited lipodystrophies are genetically predisposed from carrier parents and arise from the specific gene mutations. Inherited lipodystrophies include BSCL (Berardinelli–Seip congenital lipodystrophy) type 1–4 [4], familial partial lipodystrophy associated with mutations in PPARγ (peroxisome proliferator-activated receptor gamma) and other genes, Mandibuloacral dysplasia-type A lipodystrophy and-type B lipodystrophy.

BSCL is a rare autosomal recessive disorder, with an estimated prevalence of 1 in 10 million worldwide, discovered by Berardinelli and Seip in 1954 and 1959, respectively [5,6]. Prominent clinical features include almost complete loss of adipose tissue and a muscular appearance from birth. Affected patients exhibit accelerated growth in infancy, progressive bone ageing, fatty liver and hepatomegaly. Each BSCL type is identified by mutations in one of the four genes; *AGPAT2* (BSCL1), *Seipin* (BSCL2), *CAV1* (BSCL3) and *PTRF* (*polymerase I and transcript release factor*)*/Cavin* (BSCL4), with majority of patients (~95%) studied thus far possessing mutations in *BSCL1* or *BSCL2*. Here we will summarize the current knowledge on each of the causative genes, and focus on the mechanistic understanding of BSCL2 in this review.

## ETIOLOGIES OF BSCL

### BSCL1

Loss of ‘metabolically active’ adipose tissue is observed in BSCL1 patients, specifically at subcutaneous, intermuscular, intra-abdominal, intrathoracic and bone marrow areas [7,8]. BSCL1 patients exhibit phenotypes, including lipodystrophy, acanthosis nigricans, hepatosplenomegaly and hepatic steatosis as mentioned above. No mutations are found to be associated with neuronal dysfunction or intellectual impairment, although 10% of BSCL1 subjects (17 families/21 affected subjects) tested by Maldergem et al. showed mild or moderate intellectual impairment [9].

Aberrant expression of the *AGPAT2* (1-acylglycerol-3-phosphate-O-acyl-transferase 2) gene, on chromosome 9q34, was identified as the cause of BSCL1 [10,11]. Expression of AGPAT2 is tissue specific, high in liver, pancreas, skeletal muscle and small intestine and highest in adipose tissue [10,12]. AGPAT2 has 278 amino acids, shows up to 48% homology with AGPAT1, lower with AGPAT3-5, and catalyses the acylation of LPA (lysophosphatidic acid) to PA (phosphatidic acid) during phospholipid and TAG synthesis [13,14]. Agarwal et al. discovered ten aberrant expressions of AGPAT2 (G106fsX188, R68X, 221delGT, Q196fsX228, 140delF, L228P, L126fsX146, G136R, V167fsX183 and A239V) in 17 families [10]. Nine other genetic mutations (F60fsX102, W65X, delL165-Q196, Q172K, F109fsX452, K216X, F109fsX452, Q226X and A238G) were subsequently identified in 38 BSCL1 patients [15]. Haque et al. studied eight of the above mutants in CHO cells and reported that seven mutants exhibited significant reduction in enzyme activities, i.e. decreased acylation of LPA to PA, which could result in impaired lipogenesis [3]. However, the 8th lipodystrophy-associated A239V mutation had only mild effect on AGPAT2 enzyme activity (90% of wild-type) [3], suggesting the involvement of additional factors. Phenotypic analysis of the AGPAT2^−/−^ mouse model revealed reduced amount of WAT (white adipose tissue), mimicking observations in human counterparts [16]. Taken together, loss of AGPAT2 activity likely is the cause of BSCL1.

### BSCL2

BSCL2 patients exhibit the most severe phenotype of lipodystrophy among the four BSCLs. In addition to the loss of metabolically active adipose tissue, BSCL2 patients also exhibit loss of mechanical adipose tissue, which serves protective or supportive functions of body parts. Loss of these mechanical adipose tissue was observed in the palms, soles, scalp, orbits (around eyeball) and periarticular regions (around the joints) [7,8]. So far, predisposition of *seipin* mutations is not linked to a specific ethnic type, and BSCL2 patients studied originate from various races, including white ethnicities of Europeans, Mediterranean and Middle Eastern Arabs [9,15,17], African descendants [18] and Japanese [19].

Through a genome-wide analysis of linkage and homozygosity mapping, Magre et al. first identified *seipin* to be the responsible gene of BSCL2 in 2001 [4]. Coincidentally, in the same year Patel et al. identified a variant on chromosome 11q12–q14 in several families exhibiting a motor neuronal disorder through linkage analysis, which was termed ‘SPG17’ [20]. Windpassinger et al. then further mapped ‘SPG17’ to 11q13 and subsequently identified mutations, N88S and S90L, in *BSCL2* causing the Silver syndrome or distal hereditary motor neuropathy type V (collectively named Seipinopathy) [21,22]. In short, mutations in *seipin* are involved in two seemingly distinct disorders: lipodystrophy and motor neuropathy. It is worth noting that there is higher prevalence of cardiomyopathy and intellectual impairment in BSCL2 versus BSCL1 patients [9,23]. In fact, 78% of BSCL2 subjects (45 affected subjects in 24 families) observed by Maldergem et al. exhibit mild or moderate intellectual impairment [9], suggesting that seipin may have a direct role in the regulation of neuronal functions. Two recent studies from our laboratory support this notion by demonstrating the involvement of seipin in neurotransmission [24,25].

Although *seipin* is ubiquitously expressed, lipodystrophic mutations in *seipin* are considered loss-of-function, whereas mutations associated with motor neuropathies are deemed gain-of-function. It was originally thought that specific mutations in seipin are explicit to either lipodystrophy or seipinopathy. However, a recent finding of a novel c.985C>T nonsense mutation that results in Y289LfsX64 (tyrosine 289 mutated to leucine with 64 amino acid frameshift including stop codon) revealed a lipodystrophic phenotype, severe neuronal damage and early death at 7–8 years of age in both compound heterozygous (n=4) and homozygous children (n=2) [26]. Physiological defects include failed motor skills, myoclonic seizures and impaired cognitive development that were attributed to atrophy of the caudate nucleus (within the basal ganglia controlling motor control), posterior corpus callosum and parasagital parietal cortex. Intriguingly, cause of death was respiratory related in four out of the six subjects, either involving infection or status epilepticus. One subject was only 3.5 years of age and the cause of death of the 6th subject was not available. This study highlights that specific mutations in seipin are not necessarily explicit to lipodystrophy or Seipinopathy, but that certain mutations may cause both lipodystrophic and neuropathic manifestations.

The majority of identified mutations in seipin are nonsense, producing truncated protein products, with only a handful being missense. As such, it is reasonable that literatures to date have generally associated aberrant seipin expression to be loss-of-function mutations.

### BSCL3

A recent study by Kim et al. linked BSCL mutation of the scaffolding protein Cav1 (Caveolin-1) [27]. The nonsense mutation p.Glu38X in *Cav1* (7q31), caused by the 112G◇T nucleotide change, was found in a homozygous Brazilian female patient [27]. Intriguingly only homozygous mutations in *CAV1* exhibit a typical BSCL phenotype, with the exception of one heterozygous patient carrying the -88delC frameshift mutation, who exhibits partial lipodystrophy of upper body subcutaneous fat [28]. As no mutation of *AGPAT2* or *seipin* was found in the subject, *CAV1* was suggested as the causative gene of BSCL3. The affected patient exhibits near total loss of subcutaneous and visceral adipose tissue depots, muscular hypertrophy, hypertriglyceridemia, hepatosplenomegaly, hepatic steatosis and severe insulin resistance, typical of BSCL phenotype. CAV1 is a major structural component of caveolae, flask-shaped invaginations in the plasma membrane involved in endocytosis, lipid regulation and signal transduction [29]. The ability of CAV1 to bind to fatty acids and associate with LDs (lipid droplets) can potentially reduce uptake of fatty acids into cells and LD size if dysfunctional [30–33]. Reminiscent of patient clinical features, CAV1 null mice show reduced subcutaneous and intra-abdominal fat, adipocyte hypertrophy and interestingly, high susceptibility to tumourigenesis [34–36]. These mice also exhibit acquired lipodystrophy-like phenotype with hypertriglyceridemia, insulin resistance and resistance to diet-induced obesity [37,38]

### BSCL4

BSCL4 [39–41] was identified in consanguineous Oman families and caused by mutations in the *PTRF*/*cavin-1* gene essential for caveolae formation [41,42]. Four patients were genotyped as p.K233fs homozygous while one was heterozygous for p.E176fs mutation in PTRF. All patients exhibit typical generalized lipodystrophy, muscular hypertrophy and elevated serum creatine kinase, but vary with other phenotypes such as hepatosplenomegaly and acanthosis nigricans [42]. What is intriguing is the observation of muscular dystrophy on top of muscular hypertrophy, a potential indication of multiple roles of PTRF [39]. Causative genes of both BSCL3 and BSCL4 are somewhat related in that both CAV1, a structural protein, and PTRF/cavin-1, a transcription factor, are essential players of caveolae formation [43,44].

## MOLECULAR MECHANISMS OF SEIPIN FUNCTIONS

In the following sub-sections, we will discuss the current understanding of the molecular functions of seipin and potential pathogenic mechanisms of seipin in lipodystrophy.

### Molecular structure of seipin

**Figure 1:**
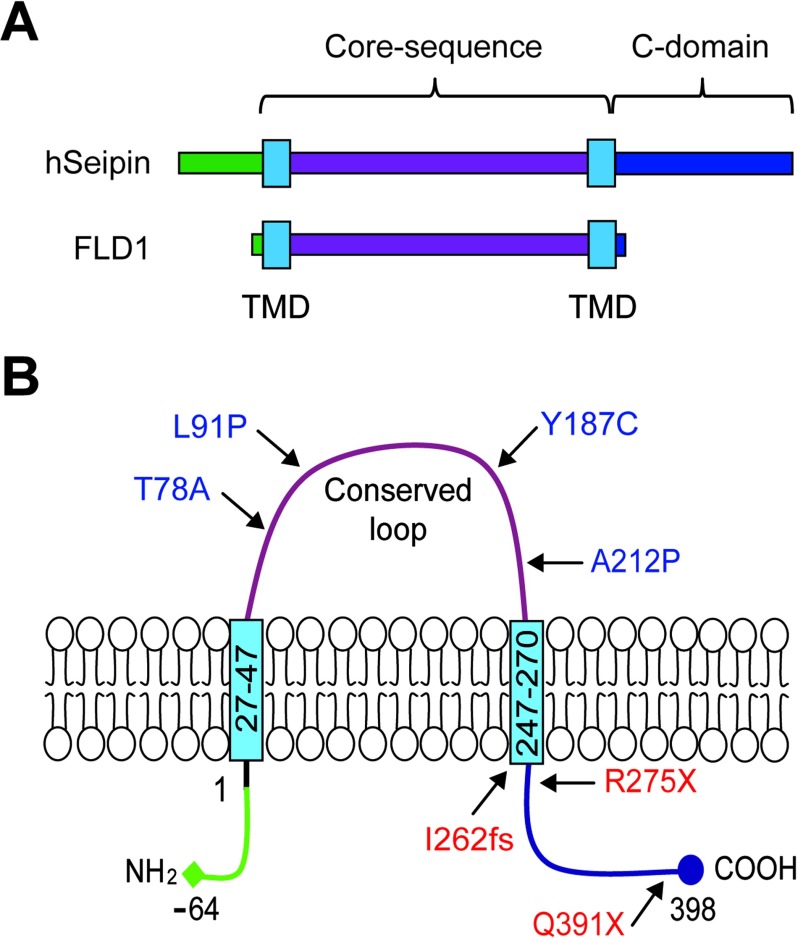
Schematic diagrams of seipin structures (**A**) Domain structure of human seipin and yeast orthologue FLD1 and (**B**) protein structure of human seipin with both N- and C- termini in the cytosol, two TMDs embedded in the ER membrane and a conserved loop localized in the ER lumen.

The structure of seipin has been well reviewed by Cartwright and Goodman [45]. *Seipin* is located on chromosome 11q13 and exists in three isoforms. Isoform 1 has 11 exons and translates to 462 amino acids. Isoform 2 lacks exon 1 and translates a shorter product of 398 amino acids without the 64 amino acids at the N-terminus, whereas isoform 3 splices out exon 7 and generates an even smaller 287 amino acid product with a shortened C-terminus [45]. All three isoforms contain two hydrophobic amino acid regions predicted as TMDs (transmembrane domains), indicating that seipin can be membrane-anchored via these sites (Figure 1A) [4]. Indeed, seipin was shown to be a resident protein of the ER (endoplasmic reticulum) where two TMDs are embedded in the ER membrane, with an evolutionary conserved domain loop in the ER lumen, and a N- and C- terminal domains exposed in the cytoplasm (Figure 1B) [22,46–48]. Orthologues of *seipin* in mammals, fruit fly and yeast contain a conserved central domain, including the TMDs and the central loop, of about 230 amino acids [45]. Although human and mouse orthologues exhibit high similarity in sequence, the yeast counterpart YLR404W/FLD1 has only 20% similarity to human and mouse *seipin*, and lacks both N- and C- termini (Figure 1A) [48].

The majority of *seipin* mutations associated with BSCL2 are nonsense or frameshift mutations that result in early truncated non-functional products, except for Q391X [18], I262fs [49] and R275X [19] in isoform 2 (Figure 1B). Q391X results in partial C-terminal truncation, whereas I262fs and R275X cause near complete loss of the C-terminal end. Importantly, these mutants retain the conserved loop region and the two TMDs. Several seipin point mutants due to missense mutations may cause misfolding of the secondary structure of the proteins. The few missense mutations involved in BSCL2 include A212P [4], T78A [18], L91P [18] and Y187C [50]. Two other missense mutations, N88S and S90L [22] are implicated in Seipinopathy. To date, most *in vivo* and *in vitro* studies investigating the function of seipin utilize wild-type, null or A212P mutant expression, with the exception of recent publication using T78A and L91P *in vitro* [51]. Seipin was proposed to be a homo-oligomer consisting of nine subunits, at least in yeast, and the wild-type protein is able to interact with its missense (N88S, S90L and A212P) mutants [52,53].

### Role of seipin in adipogenesis

As lipodystrophy is characterized by loss of adipose depots, it is necessary to study seipin in the context of adipose development. Adipogenesis is the differentiation process of a lineage committed pre-adipocyte to a fully matured adipocyte that functions as an energy storage depot in the form of TAG in LDs, governed by various adipogenic and lipogenic genes, including *PPARγ*, *C/EBPα* and *SREBP1c*. The involvement of seipin was suggested by its concordant increase in mRNA expression levels during the adipogenesis process in 3T3-L1 murine preadipocytes and C3H10T1/2 murine multipotent stem cells [54,55]. To decipher the molecular mechanism of seipin, Chen et al. isolated MEF (mouse embryonic fibroblasts) from BSCL2^−/−^ mice, which exhibit lipodystrophy, and subjected them to adipogenesis. Unexpectedly these MEF cells were able to initiate early phase adipogenesis and LD formation, and showed elevated expression of adipogenic genes *PPARγ* and *C/EBPα* in the absence of seipin. However, they lacked the ability to sustain the developmental process to fully maturate into functional adipocytes [56]. This was evident from the drastic drop in mRNA and protein expression of the adipocyte marker, *aP2* and loss of LD in mutant MEF at the terminal stage of adipocyte differentiation. The latter phenotype is consistent with other studies including ours that used seipin knockdown in C3H10T1/2 cells or the 3T3-L1 cell line, and demonstrated significant adipogenic defects accompanied by reduced mRNA levels of *PPARγ C/EBPα* and *SREBP1c* at the late stage of adipogenic induction [48,54,55]. Unexpectedly, treatment with the PPARγ agonist Pio (Pioglitazone) failed to rescue this defect in seipin knockout MEF cells [56]. This is in contrast with the previous finding showing partial restoration of adipogenesis *in vitro* and *in vivo* with treatment of thiazolidinediones compounds, rosiglitazone or Pio [55,57]. When overexpressed in 3T3-L1 cells, the seipin A212P mutant also showed inhibition of adipogenesis similar to seipin knockdown, and the impaired adipogenesis was partially restored with Pio treatment [58]. The conflict results may be due to the indirect association of PPARγ with seipin-related cellular pathways.

**Figure 2:**
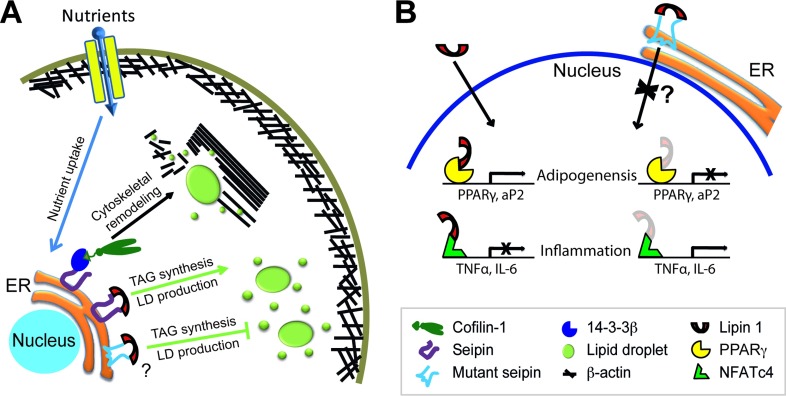
Hypothetical models of seipin functions (**A**) Uptake of excess nutrients leads to sensing of lipid levels by seipin and its recruitment of 14-3-3β and subsequently cofilin-1, inducing cytoskeleton remodelling of the cell towards adipogenesis and LD expansion. In addition, interaction with lipin 1 at the ER is involved in TAG synthesis and storage in LDs. (**B**) Activities of lipin 1 in the nucleus in adipogenesis and inflammation. Seipin point mutants such as A212P, still retaining interactive capability to lipin 1 and 14-3-3β, mis-localize to the nuclear region. The complex may sequester lipin 1 co-activator activity with PPARγ or 14-3-3β-binding partner cofilin-1 and suppress adipogenesis, and/or sequester lipin 1 co-repressor activity with NFATc4, causing up-regulated expression of inflammatory cytokines TNFα and IL-6.

It appears that the C-terminus is indispensible for seipin to regulate adipogenesis, in cooperation with the evolutionarily conserved core sequence as a potential lipid sensor [48]. This regulation was shown to involve the direct interaction of seipin C-terminus with 14-3-3β, which in turn recruits cofilin-1 to remodel actin cytoskeleton during adipocyte development (Figure 2A) [59]. This model explains how C-terminal truncation leads to lipodystrophy, such as in the case of patients with I262fs, R275X and Q391X mutations [18,19,49]. However, it is unclear why the missense mutations in the loop region lead to lipodystrophy, such as A212P, which still retain the C-terminal sequence (Figure 1B). One possible mechanism is that the A212P point mutation may be associated with activation of inflammation pathways during pre-adipocyte stage [58] (see below for more). An alternative model may be that the A212P mutation leads to disruption of secondary protein structure and thus inability of the core sequence to sense lipid level, as the mutant protein fails to inhibit LD formation in preadipocytes [48,59]. It is interesting to note that A212P inhibition of adipogenesis was also suggested to be an overexpression artifact [60]. However, we observed no significant inhibition in adipogenesis when seipin mutants unrelated to lipodystrophy, such as Seipinopathy mutants N88S and S90L, were overexpressed in 3T3-L1 cells. Both N88S and S90L mutations, which disrupt N-glycosylation of seipin, interact with the ER chaperone calnexin due to mutations in a glycosylation site and was proposed to activate ER stress response or autophagy [61,62]. The difference in adipogenic effects by A212P versus N88S/S90L warrants further investigation to determine to what extent the structural disruption of mutant proteins can account for the lipodystrophic effects.

### Role of seipin in LD formation and lipid homoeostasis

To understand the functions of seipin and the underlying molecular mechanisms, it is critical to differentiate the studies on lipogenesis and LD formation from those on adipogenesis. Although lipogenesis and LD formation can happen in many different cell types, adipogenesis is only applicable to adipocytes that are specialized in storing lipids. Here we discuss the functions of seipin in non-adipocytes (including yeast, hepatocytes and undifferentiated 3T3-L1 and other preadipocytes treated with oleate) and adipocytes (preadipocytes treated with adipogenic cocktail and mature adipocytes).

#### In non-adipocytes

In 2008, Fei et al. showed that in yeast, depletion of a putative seipin orthologue FLD1 (FLD1Δ) exhibited LD-associated defects [63]. These defects include altered quantity and size of LDs in FLD1Δ cells, with ~30% of mutant cells containing one or a couple of ‘supersized’ LD (0.5–1.5 μm as opposed to 0.2–0.4 μm in wild-type cells), suggesting fusion of small droplets in FLD1-deficient cells. Higher amounts of TAG and sterol esters were also accumulated in the LDs of FLD1Δ cells, consistent with inhibition of lipolysis in FLD1Δ yeast reported by another group [64]. Interestingly, the defects observed in yeast could be rescued by expression of human Seipinopathy mutants N88S and S90L, but not lipodystrophy-associated A212P mutant [63]. However, when 3T3-L1 cells expressing N88S and S90L mutants were treated with oleate to induce LD formation and lipid accumulation, small LDs were observed instead, a phenotype reminiscent of seipin knockdown [53]. Similar to FLD1Δ yeast cells, seipin knockdown 3T3-L1 preadipocytes exhibited enhanced TAG synthesis. The observed increase in TAG synthesis in non-adipocytes was further confirmed in seipin knockout mice on chow diet, which exhibited elevated TAG content in the liver [56,57]. In contrast, A212P overexpression did not result in such an enhanced LD formation and exhibited a phenotype similar to normal 3T3-L1 cells [53]. Consistent with the latter study, we did not observe defective LD formation when A212P seipin expressing 3T3-L1 cells were overloaded with oleate, suggesting that there is no defect in TAG storage. Surprisingly, overexpression of wild-type seipin specifically inhibits TAG synthesis and LD formation in non-adipocytes, which may not involve enhanced lipolysis [48,53,59]. Since seipin is composed of a conserved loop in the ER lumen and the C-terminus in the cytoplasm, we truncated seipin either by removing the C-terminus [ΔCT] or the loop domain [CT] [26,48]. Importantly, we found significant inhibition of LD formation when ΔCT or wild-type seipin overexpressing cells were subjected to oleate treatment. This is in stark contrast to the effect of overexpressing CT or A212P seipin where LD formation was unaffected. Although seipin A212P plays an inhibitory role in adipogenesis programming, this mutation does not seem to affect TAG accumulation in non-adipocytes, which may account for the observation of TAG storage in non-adipose organs in BSCL2 human patients. As mentioned above, the A212P mutant may function as a dominant negative by interacting with seipin-binding partners through its cytosolic N- or C-termini, thus sequester and prevent the binding partners from interacting with endogenous wild-type seipin when A212P mutant is overexpressed.

A recent interesting study examined the relations of seipin and HCV (hepatitis C virus). By overexpressing wild-type seipin in a hepatocellular carcinoma cell line Huh7, the authors showed increased size and reduced number of LDs per cell. This led to reduced total outer surface area of LDs, which was correlated with reduced production of HCV particles [65]. This finding suggests a possibility that expression of seipin mutants may affect HCV, at least *in vitro*, and potentially increase the risk for liver cirrhosis and hepatocellular carcinoma in BSCL2 patients.

#### In adipocytes

In BSCL2^−/−^ MEF cells during adipogenesis, phosphorylation of hormone-sensitive lipase and perilipin 1A, target substrates of PKA (protein kinase A), is increased [56]. It was proposed that this enhanced lipolysis activity is the result of failed adipogenesis, although the underlying mechanism is unclear. Indeed, treatment of H89 (PKA inhibitor) or E600 (TAG lipase inhibitor) could rescue the adipogenic defect in BSCL2^−/−^ MEF cells, as evidenced by elevated levels of *C/EBPα*, *PPARγ* and *aP2* [56]. Another potential mechanistic model is the plausible reduction in TAG synthesis through interaction and modulation of lipin 1, a PA phosphatase, by the C-terminus of seipin [66]. Levels of PA, a substrate of lipin 1, were significantly increased upon seipin knockdown, and decreased by seipin overexpression in differentiating 3T3-L1 cells, indirectly signifying the reduced and enhanced TAG synthesis, respectively. Besides its enzymatic activity in TAG synthesis, lipin 1 also regulates transcriptional activities as a co-activator or co-repressor of transcription factors. Lipin 1 activity is indispensable in 3T3-L1 adipogenic programming through the co-activation of the transcription factor PPARγ, as evidenced by reduced TAG storage and suppressed expression of adipogenic markers upon its knockdown [67]. Considering the observation that seipin mutations L91P and A212P both retain the binding capability to lipin 1 and mis-localize to the nuclear envelope, it is reasonable to propose that both of these mutants may contribute to the transcription activity of lipin 1 leading to suppression of its target PPARγ (Figure 2B) [51,54].

Three *in vivo* models thus far have highlighted the differential roles of seipin in LD formation in non-adipocytes and adipocytes. Knockdown of seipin leads to (1) enhanced TAG synthesis and LD formation in non-adipocytes, and (2) dysfunction of adipocyte development and LD formation through enhanced lipolysis or inhibition of TAG production. While the absence of seipin expression evidently inhibits LD development during adipogenesis, excess expression of wild-type seipin appears to have a similar detrimental effect. In a mouse model overexpressing 150% more seipin in adipose tissue (aP2-seipin), MRI and histological analysis showed significant reduction in subcutaneous and intra-abdominal fat and up to 50% increase in TAG storage in the liver [68]. Instead of adipocyte development, excessive expression of seipin increased the rate of lipolysis similar to the lipolysis model found in knockout mice above [56,68]. The precise mechanism underlying the phenotype remains elusive.

Taken into consideration of our recent studies that seipin regulates lipid homoeostasis by preventing lipid overloading in non-adipocytes while promoting lipid storage in adipocytes [48], and that seipin binds to 14-3-3β through the cytosolic N- and C-termini of seipin [59], a plausible model for the function of seipin in adipogenesis starts to emerge: excess nutrients stimulate the uptake of lipids into preadipocytes during adipogenesis, and the accumulation of lipids in the ER is sensed by seipin; this energy storage signal is transduced to the cytoplasm via seipin-interacting partner 14-3-3β, which mediates the recruitment of cofilin-1, thereby coordinating the remodelling of actin cytoskeleton to accommodate LD formation and expansion in adipocytes (Figure 2A). Presumably there are other binding partners associated with 14-3-3β in regulating multiple cellular pathways to promote adipogenesis. Interestingly, a recent study showed that seipin binds and modulates activity of SERCA (sarco/endoplasmic reticulum Ca^2+^-ATPase) that transport calcium from cytosol to ER lumen [69]. Although intracellular calcium gradient appears to signal for lipid storage, further studies are necessary to understand how this cooperates with the above proteins and contributes to adipogenesis in mammalian cells.

### Seipin and inflammation

Among potential pathological features of CGL/BSCL is the inflammatory activation. So far no report has yet to tie BSCL disorders to inflammation, although there was a report of elevated circulating TNFα (tumour necrosis factor α) levels in patients of HIV-associated lipodystrophy [70]. Additionally, in a relatively new syndrome identified as CANDLE (‘chronic atypical neutrophilic dermatosis with lipodystrophy and elevated temperature’) four patients exhibited lipodystrophic phenotype similar to that seen in acquired partial lipodystrophy [71]. Systemic inflammation was observed, with an elevated erythrocyte sedimentation rate (an indirect measure of inflammation), increased C-reactive protein (produced by liver in response to inflammatory cytokines) and dermal infiltration of mature neutrophils and mononuclear myeloid cells. Although preliminary, the authors speculated that lipodystrophy could be a result of elevated secretion of inflammatory cytokines IL-6 (interleukin-6) and TNFα. Recent findings from our laboratory revealed a significant elevation of inflammatory pathways involving TNFα, IL-6, iNOS (inducible nitric oxide synthase), COX2 (cyclooxygenase-2) and MCP-1 (monocyte chemoattractant protein-1) when A212P seipin overexpressing 3T3-L1 cells were subjected to adipogenesis [58]. Similarly, elevated circulating and mRNA levels of TNFα, IL-6 and MCP-1 in WAT and brown adipose tissue were found in a transgenic mouse model overexpressing nSREBP1c (nuclear SREBP1c) that mimics lipodystrophy [72]. MCP-1 recruits macrophages to adipose tissue and induces an inflammatory response, which was observed in this nSREBP1c lipodystrophy model.

Adipose tissue macrophage infiltration and inflammatory response are often associated with an obesity phenotype, where enlarged adipocytes release free fatty acids through lipolysis and pro-inflammatory cytokines TNFα, IL-6 and MCP-1, resulting in development of insulin resistance. For instance, IL-6 inhibits insulin receptor substrate 1, impairs insulin stimulated glucose transport, and causes insulin resistance [73,74]. Results from our laboratory showed that treatment of TUDCA (tauroursodeoxycholic acid), a stabilizer of protein misfolding, or Indomethacin, an anti-inflammatory drug, could significantly rescue the adipogenic defects in seipin A212P expressing 3T3-L1 cells [58]. Although the precise mechanism was not clear, IL-6 was significantly reduced by TUDCA treatment in the mutant cells, thus providing a potential therapeutic approach of BSCL2. While the inflammatory mechanism of lipodystrophy is relatively a new idea, overlapping aetiology between lipodystrophy and obesity might indicate that a platform of obesity treatment may well be applied to BSCL2 treatment. Indeed administration of chemical chaperons, TUDCA and 4-PBA in mice fed with high-fat diet showed significant reduction in both ER stress and inflammation in adipose tissue and reversal of impaired insulin signalling induced by the diet [75]. A hypothetical inflammatory model may be again linked to lipin 1. As mentioned above, lipin 1 serves as a co-activator of PPARγ transcription activity. Conversely, lipin 1 can additionally act as a co-repressor of the transcription factor NFATc4 (nuclear factor of activated T cells c4), and down-regulate expression of inflammatory cytokines IL-6 and TNFα in 3T3-L1 adipocytes [76]. Furthermore, MCP-1 expression and secretion increased when lipin 1 expression was suppressed in 3T3-L1 adipocytes, presumably via NFATc4 activities [77]. Together, it is plausible that mutant seipin mislocalization to the nuclear envelope along with its association with lipin 1 may inhibit the suppressive activity of lipin 1 on NFATc4, leading to subsequent activation of inflammation in lipodystrophy, as also observed in obesity associated inflammation (Figure 2B) [76]

## CONCLUDING REMARKS

The role of BSCL2/seipin seems to be complex and studies to date suggested that this enigmatic protein may differentially regulate lipid homoeostasis by inhibiting LD formation in non-adipocytes while promoting adipogenesis to store excess lipid. Depletion of seipin leads to increased TAG accumulation in non-adipocytes but inhibition of adipogenesis. However, expression of the seipin mutant, specifically A212P, has no effect on TAG accumulation in non-adipocytes yet inhibits adipogenesis. Mechanistically, the C-terminus of seipin appears to play an essential role in both LD biogenesis and adipogenic programming, shedding light on the pathogenesis of non-functional truncated protein due to nonsense mutations, such as R275X, I262fs and Q391X, which lack the C-terminus. However, other point mutations, T78A, L91P and A212P, seem to retain the C-terminal region. In our model (Figure 2), the conserved core sequence has its distinct function in sensing lipid levels, either preventing lipid accumulation in non-adipocytes or facilitating lipid accumulation in adipocytes. This scenario may possibly explain why A212P and other missense mutants with C-terminus cause lipodystrophy. Nevertheless, seipin mutations in the loop domain may lead to other cellular responses to inhibit adipogenesis. Our study proposes another possible mechanism of inflammatory activation as seen in HIV-lipodystrophy and CANDLE. Further studies are necessary to address validity of these scenarios by identifying other novel molecular players and delineating cellular pathways that differentially regulate LD biogenesis and lipogenesis in non-adipocytes and adipocytes.
